# Association of High-Density Lipoprotein Cholesterol With GFR Decline in a General Nondiabetic Population

**DOI:** 10.1016/j.ekir.2021.05.007

**Published:** 2021-05-19

**Authors:** Toralf Melsom, Jon Viljar Norvik, Inger Therese Enoksen, Vidar Stefansson, Renathe Rismo, Trond Jenssen, Marit D. Solbu, Bjørn O. Eriksen

**Affiliations:** 1 Metabolic and Renal Research Group, UiT Arctic University of Norway; 2 Section of Nephrology, University Hospital of North Norway, Tromsø; 3 Department of Organ Transplantation, Oslo University Hospital and University of Oslo, Norway

**Keywords:** aging, chronic kidney disease, GFR, glomerular filtration rate, high-density lipoprotein cholesterol, HDL cholesterol

## Abstract

**Introduction:**

Although lower high-density lipoprotein cholesterol (HDL-C) levels are considered a risk factor for cardiovascular disease (CVD), experimental evidence suggest that aging, inflammation, and oxidative stress may remodel HDL-C, leading to dysfunctional HDL-C. Population studies on HDL-C and loss of the glomerular filtration rate (GFR) reported inconsistent results, but they used inaccurate estimates of the GFR and may have been confounded by comorbidity.

**Methods:**

We investigated the association of HDL-C levels with risk of GFR loss in a general population cohort; the participants were aged 50–62 years and did not have diabetes, CVD, or chronic kidney disease (CKD) at baseline. The GFR was measured using iohexol-clearance at baseline (*n*=1627) and at the follow-up (*n*=1324) after a median of 5.6 years. We also investigated any possible effect modification by low-grade inflammation, physical activity, and sex.

**Results:**

Higher HDL-C levels were associated with steeper GFR decline rates and increased risk of rapid GFR decline (>3 ml/min per 1.73 m^2^ per year) in multivariable adjusted linear mixed models and logistic regression (–0.64 ml/min per 1.73 m^2^ per year [95% CI –0.99, –0.29; *P* < 0.001] and odds ratio 2.7 [95% CI 1.4, 5.2; *P* < 0.001] per doubling in HDL-C). Effect modifications indicated a stronger association between high HDL-C and GFR loss in physically inactive persons, those with low-grade inflammation, and men.

**Conclusion:**

Higher HDL-C levels were independently associated with accelerated GFR loss in a general middle-aged nondiabetic population.

## Graphical abstract

**Figure:**
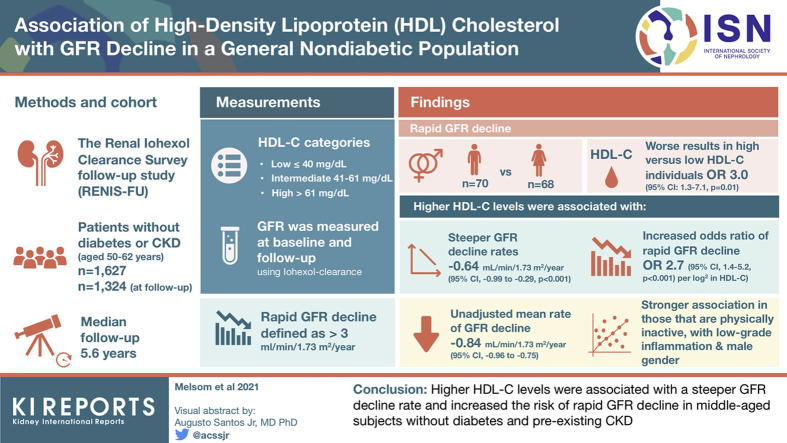

Kidney function, as assessed by the GFR, declines with age, even in healthy individuals, leading to a high prevalence of CKD in the elderly population.^1^ However, there is large variation in the rate of GFR decline among individuals, regardless of risk factors such as diabetes and hypertension.^2^^,^^3^ Although the mechanisms leading to age-related GFR decline are largely unknown, the interindividual variation in the rate of GFR decline indicates that CKD may be prevented.

Lower HDL-C levels have been considered a risk factor for atherosclerosis, CVD, and CKD for decades. This paradigm has been challenged, as clinical trials designed to increase HDL-C levels have failed to show any clinical benefits,^4^ and most Mendelian randomization studies have not confirmed low HDL-C to be a risk factor for CVD or CKD.^5^, ^6^, ^7^, ^8^ However, Mendelian randomization studies may not account for pleiotropic effects of the included genes, nonlinear associations between the risk factors and outcomes, and different or opposite effects of changes in HDL-C levels across population subgroups.^6^^,^^9^

Previous experimental studies and human data indicate that there is a complex association between higher HDL-C levels and vascular dysfunction, atherosclerosis, and kidney dysfunction.^9^ HDL-C is a large molecule with multiple potentially beneficial functions, but proinflammatory enzymes, hyperglycemia, and oxidative stress may remodel HDL-C, leading to dysfunctional and proinflammatory HDL-C particles.^9^, ^10^, ^11^, ^12^ Increased levels of dysfunctional HDL-C particles have been associated with a sedentary lifestyle, an older age, low-grade systemic inflammation, and higher risk of CVD.^9^, ^10^, ^11^, ^12^, ^13^ In the kidneys, both HDL-C deficiency and HDL-C dysfunction have been linked to vascular atherosclerosis and tubulointerstitial injury in experimental studies.^14^, ^15^, ^16^ These possible dual effects of HDL-C are in accordance with the results of epidemiologic studies showing a U-shaped association between HDL-C levels and the risk of CKD, CVD, or all-cause mortality in various populations.^17^, ^18^, ^19^

Although several studies have reported an association of low HDL-C levels with incident CKD, both low and high HDL-C levels were associated with a GFR loss, CKD progression and end-stage kidney disease in a study of nearly 2 million male US veterans.^19^ However, none of these studies fully adjusted for possible confounding factors, and they were all limited by the use of the estimated GFR (eGFR). eGFR based on creatinine or cystatin-C levels is biased by non–GFR-related factors such as muscle mass, inflammation, and obesity and may therefore lead to confounded results, particularly in studies on metabolic risk factors and in older persons.^20^, ^21^, ^22^, ^23^

In this study, we investigated the association of HDL-C levels with decline in measured GFR in persons from the general population without pre-existing CKD, diabetes, or CVD. Because physical activity, low-grade inflammation, and sex have been shown to influence HDL-C functionality,^9^^,^^12^^,^^24^^,^^25^ we also investigated any possible effect modification caused by these factors.

## Methods

### Study Population

**Figure 1:**
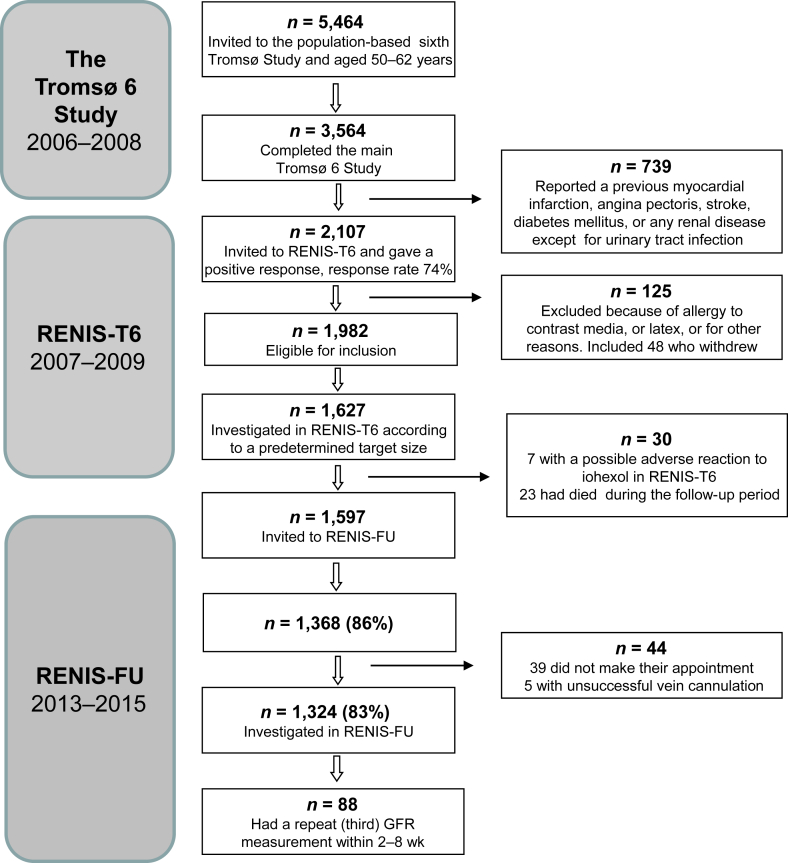
Flowchart of the Renal Iohexol clearance survey (RENIS).

The Renal Iohexol Clearance Survey (RENIS) is a substudy of the sixth wave of the population-based Tromsø Study (Tromsø 6), Northern Norway. A 40% random sample of individuals in the municipality of Tromsø aged 50–59 years and all individuals aged 60–62 years (5464 total subjects) were invited, and 3564 (65%) individuals completed the main study. Participants who did not report having a history of myocardial infarction, angina pectoris, stroke, diabetes, or kidney disease were invited to join RENIS-T6 (Figure 1). A total of 1982 subjects were eligible for inclusion, and 1627 were included in a random order according to a predetermined target for number of participants in the RENIS-T6.^21^ A follow-up measurement of the GFR in the RENIS follow-up study (RENIS-FU) was recorded for 1324 (81%) participants after a median observation time of 5.6 years (interquartile range 5.2–6.0) (Figure 1). A random sample of 88 persons participated in a second follow-up within 8 weeks after the RENIS-FU. This repeat GFR measurement conducted in a subsample allowed us to calculate the day-to-day variation in the GFR measurements and to use a linear mixed regression model in longitudinal data analyses.

The RENIS study was approved by the local ethics committees and performed in accordance with the guidelines of the Declaration of Helsinki. All subjects provided written informed consent.

### Measurements

The RENIS-T6 and RENIS-FU were conducted at the Clinical Research Unit at the University Hospital of North Norway. The participants fasted at home from midnight and were asked to drink 2 glasses of water in the morning before they came to the hospital between 8:00 am and 10:00 am to have their GFR measured and blood samples drawn. Participants with symptoms of intercurrent illness had to reschedule their appointments.

The GFR was measured at baseline and at follow-up with single-sample plasma clearance of iohexol (mGFR) as previously described in detail.^26^ This method has been validated against gold standard methods and was recently found to show substantial agreement with the multiple-sample method.^20^^,^^27^ The intraindividual coefficient of variation for the GFR measurement (day-to-day variation) was 4.2% (3.4%–4.9%).^3^

The fasting serum glucose, triglycerides, total cholesterol, low-density lipoprotein cholesterol, and HDL-C concentrations were measured by a Modular P800 (Roche Diagnostics, Mannheim, Germany) instrument. The HDL-C level was categorized as low when it was ≤1.0 mmol/l (≤40 mg/dl), intermediate when it was 1.1–1.6 mmol/l (41–61 mg/dl), and high when it was >1.6 mmol/l (>61 mg/dl), as suggested previously.^28^^,^^29^

Serum creatinine analyses were performed using a standardized enzymatic assay, and cystatin C was measured by particle-enhanced turbidimetric immunoassay.^30^ The GFR was estimated using the Chronic Kidney Disease Epidemiology Collaboration (CKD-EPI) equations.

Three samples of first-void morning spot urine were collected on consecutive days prior to the GFR measurements. The urinary albumin and creatinine concentrations were measured in fresh urine, as previously reported.^31^ The albumin-to-creatinine ratio (ACR) in mg/mmol was calculated for each urine specimen, and the median ACR value was used in the analyses.

High-sensitivity C-reactive protein (Hs-CRP) and HbA_1c_ were measured in the main Tromsø 6 study, as described previously.^30^^,^^32^

Blood pressure was measured 3 times in a seated position after a 2-minute rest period. The average of the second and third measurements was used in the analyses.

### Questionnaire

A health questionnaire included questions on tobacco and alcohol use, current medications, and physical activity related to the frequency and intensity of leisure-time physical exercise.^33^^,^^34^ The reliability for the physical activity questions was reported to be good, and the correlation between reported physical activity and maximal oxygen consumption (Vo_2max_) was moderate in a study where questions were repeated and physical fitness was assessed by the measurement Vo_2max_ (Spearman correlation and weighted kappa frequency for test-retest: *r* = 0.82–0.87, and correlation with Vo_2max_: *r* = 0.40–0.48; *P* < 0.01).^35^

We dichotomized physical activity, as reported in a previous publication, as follows: active (>1-h hard physical activity a week [becoming breathless, sweaty, or exhausted] and/or >3-h light activity [without becoming breathless or sweaty]) or inactive (all others).^33^

Alcohol use was categorized according to the frequency at which subjects drank alcohol (never, once a month or less, 2–4 times a month, 2–3 times a week, or >4 times a week). Individuals were categorized as being a daily smoker, previously being a daily smoker, or never being a daily smoker.

### Statistical Methods

A linear trend across groups by HDL-C levels was tested with linear or median regression for continuous variables and with logistic regression for dichotomous variables. The association between the baseline HDL-C levels (as a categorical and log-transformed continuous variable) and change in the GFR was analyzed by a linear mixed regression model with a random intercept and slope. All 1627 participants with 1 to 3 GFR measurements were included in the analyses because linear mixed models allow for missing observations at 1 or more time points as long as the observations are missing at random.^36^^,^^37^ Missing of the third GFR measurement for the majority of participants was part of the design of this study, and these observations are “missing completely at random.” For the minority of subjects who did not have a follow-up measurement it is plausible that they are missing at random conditional on the baseline variables. Although 3 measurements were only available for a random subsample (*n*=88) in the RENIS-FU, this method allowed us to estimate the 3 variance components in the unstructured covariance matrix of the model. The association of the HDL-C level with the rate of GFR decline was analyzed by including 2-way interaction terms between the HDL-C variable and the time variable.

The association of HDL-C with the odds of rapid GFR decline was analyzed using logistic regression for those with at least 1 follow-up (*n* = 1324). Rapid GFR decline was defined as a rate of GFR decline steeper than 3 ml/min per 1.73 m^2^ per year (calculated as GFR_follow-up_ – GFR_baseline_ / observation time), a cut-off that has been used in previous studies.^38^^,^^39^ In sensitivity analyses, we defined the subjects with rapid GFR decline as the 10% of subjects with the steepest rates of GFR decline, as calculated using an adjusted linear mixed model.^40^^,^^41^

In the linear mixed regression models, we adjusted for baseline variables that are known or assumed to be associated with HDL-C levels and GFR loss in 3 separate models: for model 1, age and sex; for model 2, model 1 + body mass index, fasting triglycerides, the use of lipid-lowering drugs, and alcohol consumption; for model 3, model 2 + systolic blood pressure, low-density lipoprotein cholesterol level, fasting glucose level, smoking status, leisure-time physical activity, waist-to-hip ratio, hs-CRP level, ACR, and the use of antihypertensive medications. In the logistic regression analyses, we included a fourth model with an additional adjustment for the baseline GFR.

We tested for effect modification by age, sex, hs-CRP level, and physical activity by including an interaction term between each of these variables and HDL-C and, in the linear mixed regression models, a triple interaction term that also included the time variable. Nonlinear associations between HDL-C and GFR decline were investigated by including a quadratic term for HDL-C.

The statistical significance level was set to be 0.05. All statistical analyses were performed in Stata/MP 16.0 (Stata Corp., College Station, TX).

## Results

**Figure 2:**
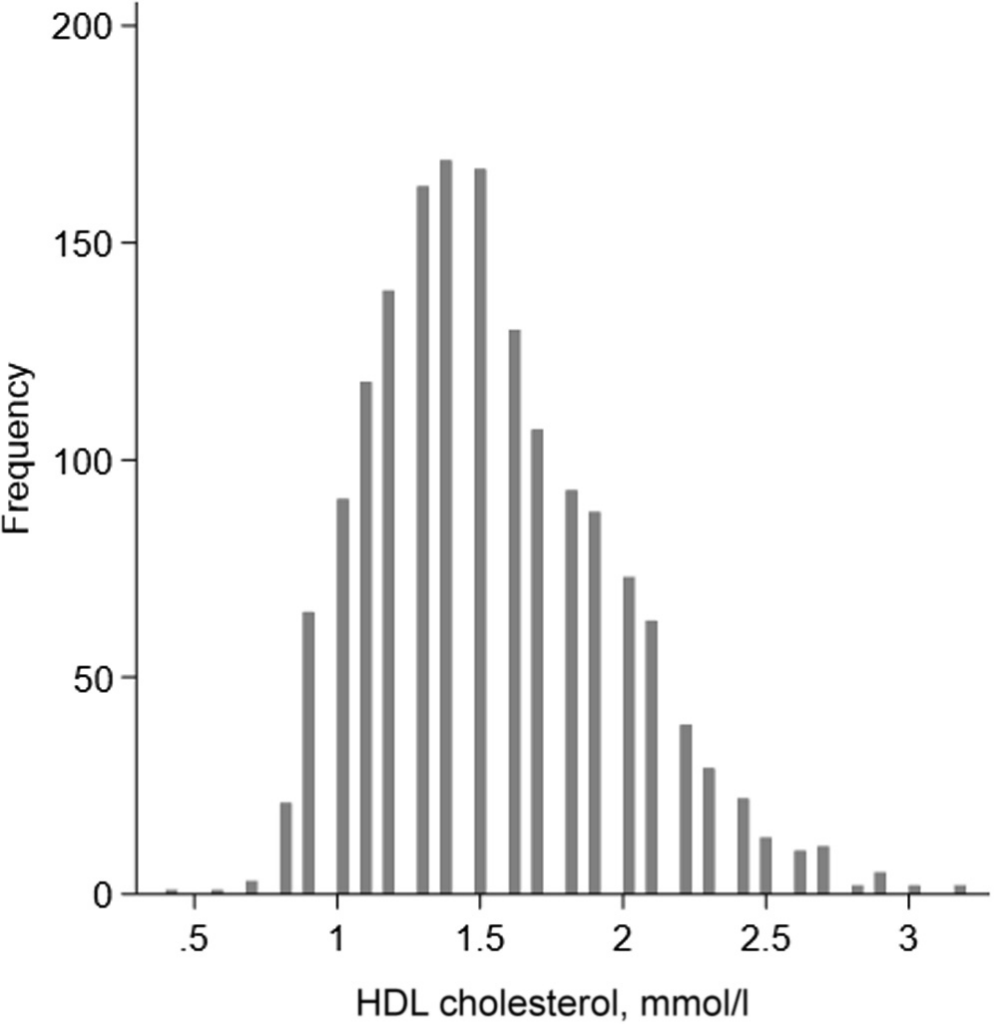
The distribution of high-density lipoprotein cholesterol (HDL-C) levels at baseline.

The study population characteristics at baseline grouped by low, intermediate, and high levels of HDL-C are shown in Table 1 and by sex-specific quartiles of HDL-C in Supplementary Table S1. Fifty-one percent (*n* = 826) were women, the mean (SD) age was 58.1 (3.8) years, and the mean GFR was 93.9 (14.4) ml/min per 1.73 m^2^. The median HDL-C level was 1.5 (interquartile range 1.2–1.8) mmol/l (58 [interquartile range 46–70] mg/dl). The distribution of HDL-C levels at baseline is shown in Figure 2. Participants with higher HDL-C levels were more often women and generally had a healthier risk profile, but they consumed alcohol more often (Table 1).

**Table 1:** Study population at baseline by HDL-C levels

Characteristics	Overall (*N* = 1627)	Low HDL-C (≤1.0 mmol/l) (*n* = 182)	Intermediate HDL-C (1.1–1.6 mmol/l) (*n* = 886)	High HDL-C (>1.6 mmol/l) (*n* = 559)	*P* value
Women, *n* (%)	826 (51)	42 (23)	386 (44)	398 (71)	<0.001
Age, yr	58.0 (3.8)	58.2 (53.9–61.2)	58.5 (54.6–61.3)	59.0 (54.9–61.7)	0.02
Body mass index	27.3 (4.0)	28.6 (26.6–31.2)	27.5 (25.4–30.3)	25.0 (22.9–27.8)	<0.001
Waist-hip ratio	0.91 (0.07)	0.96 (0.92–1.03)	0.92 (0.88–0.97)	0.87 (0.83–0.92)	<0.001
Systolic blood pressure, mm Hg	130 (18)	129 (121–142)	130 (118–142)	126 (113–138)	<0.001
Diastolic blood pressure, mm Hg	83 (10)	84 (79–91)	84 (78–91)	81 (74–88)	<0.001
Blood pressure medication, *n* (%)	299 (18)	44 (24)	186 (21)	69 (12)	<0.001
Fasting blood glucose, mmol/l	5.4 (0.6)	5.4 (5.1–5.8)	5.3 (5.1–5.7)	5.1 (4.9–5.5)	<0.001
Total cholesterol, mmol/l	5.6 (0.9)	5.4 (4.7–6.3)	5.6 (5.0–6.2)	5.6 (5.2–6.3)	<0.001
LDL-C, mmol/l	3.7 (0.9)	3.8 (3.2–4.4)	3.7 (3.2–4.3)	3.4 (2.9–4.0)	<0.001
HDL-C, mmol/l	1.5 (1.2–1.8)	0.95 (0.90–1.00)	1.4 (1.2–1.5)	1.9 (1.8–2.1)	
Triglycerides, mmol/l	1.0 (0.8–1.5)	1.8 (1.3–2.4)	1.1 (0.9–1.5)	0.8 (0.6–1.0)	<0.001
Lipid-lowering medication, *n* (%)	107 (7)	9 (5)	62 (7)	36 (6)	0.6
High-sensitivity CRP, mg/l	1.20 (0.65–2.26)	1.64 (0.99–3.47)	1.34 (0.70–2.43)	0.93 (0.51–1.65)	<0.001
Daily smoker, *n* (%)					0.01
Never	504 (31)	51 (28)	268 (30)	185 (33)	
Yes, previously	771 (47)	76 (42)	437 (49)	258 (46)	
Yes, currently	344 (21)	52 (29)	180 (20)	112 (20)	
Alcohol use, *n* (%)					<0.001
Once a month or less	461 (28)	79 (43)	255 (29)	127 (23)	
2–4 times a month	717 (44)	80 (44)	402 (45)	235 (42)	
2 times a week or more	442 (27)	23 (13)	225 (25)	194 (35)	
Physical activity^b^, *n* (%)					
>1-h high-intensity and/or >3-h low-intensity per week	705 (43)	70 (38)	358 (40)	277 (50)	0.01
Urinary ACR, mg/mmol	0.23 (0.10–0.54)	0.31 (0.10–0.54)	0.23 (0.10–0.53)	0.22 (0.10–0.56)	0.01
mGFRiohexol, ml/min per 1.73 m^2^	93.9 (14.4)	96.5 (86.4–106.3)	94.7 (85.6–104.2)	92.0 (84.3–101.2)	<0.001
mGFR_Follow-Up_, ml/min per 1.73 m^2^	89.0 (14.5)	92.6 (82.6–102.2)	89.8 (80.8–100.0)	87.5 (77.7–95.9)	<0.001
eGFR_CKDEPIcrea_, ml/min per 1.73 m^2^	94.8 (9.5)	97.3 (92.7–101.5)	97.1 (90.1–101.3)	96.1 (90.4–100.5)	0.3
eGFR_CKDEPIcrea FU_, ml/min per 1.73 m^2^	88.2 (10.5)	90.6 (82.1–96.1)	91.1 (83.2–95.5)	90.0 (82.1–95.0)	0.2
Follow-up time, yr	5.6 (5.2–6.0)	5.7 (5.1–6.0)	5.6 (5.2–6.0)	5.7 (5.3–6.0)	

ACR, albumin-to-creatinine ratio; CRP, C-reactive protein; HDL-C, high-density lipoprotein cholesterol; LDL-C, low-density lipoprotein cholesterol; mGFRiohexol, the glomerular filtration rate measured using iohexol clearance.

Data are presented as mean (SD) and median (interquartile range) for continuous variables and *n* (%) for dichotomous variables.

b Based on self-reported leisure-time physical activity: Active (>1-h hard physical activity a week [becoming breathless or sweaty, or exhausted] and/or >3-h light activity [without becoming breathless or sweaty]) or inactive (all others).^33^

### Association of HDL-C With GFR Decline

The unadjusted mean rate of GFR decline was –0.84 (95% CI –0.96, –0.75) ml/min per 1.73 m^2^ per year. A rapid GFR decline (GFR loss > 3.0 ml/min per 1.73 m^2^ per year) was observed in 70 men and 68 women. Higher HDL-C levels were associated with a steeper annual GFR decline and an increased odds ratio (OR) of rapid GFR decline (beta coefficient: –0.64 ml/min per 1.73 m^2^ per year (95% CI –0.99, –0.29; *P* < 0.001) and OR 2.7 (95% CI 1.4, 5.2; *P* < 0.001) per doubling (log2) in HDL-C) (Tables 2 and 3). Subjects with HDL-C >1.6 mmol/l had an odds ratio of 3.0 (95% CI 1.3, 7.1; *P* = 0.01) for rapid GFR decline, compared to subjects with HDL-C ≤1.0 mmol/l. There was no relevant collinearity between covariates (mean and maximum variation inflation factor was 1.3 and 2.2), and the logistic regression models were well calibrated according to the Hosmer-Lemeshow statistics.

**Table 2:** Association between baseline HDL-C levels and annual GFR change rates

	Model 1			Model 2			Model 3		
	GFR, ml/min per 1.73 m^2^ per year^a^	(95 % CI)	*P* value	GFR, ml/min per 1.73 m^2^ per year^a^	(95 % CI)	*P* value	GFR, ml/min per 1.73 m^2^ per year^a^	(95 % CI)	*P* value
HDL-C, per doubling (log2)	–0.22	(–0.51, 0.06)	0.13	–0.53	(–0.87, –0.18)	<0.01	–0.64	(–0.99, –0.29)	<0.001
Low HDL-C^b^	Ref			Ref			Ref		
Intermediate HDL-C^b^	–0.11	(–0.46, 0.23)	0.53	–0.32	(–0.70, 0.06)	0.10	–0.29	(–0.67, 0.10)	0.15
High HDL-C^b^	–0.20	(–0.57, 0.18)	0.30	–0.51	(–0.95, –0.08)	0.02	–0.53	(–0.97, –0.08)	0.02

GFR, glomerular filtration rate; HDL-C, high-density lipoprotein cholesterol.

Model 1: Adjusted for sex and age.

Model 2: Model 1 + body mass index, triglycerides, use of lipid-lowering drugs, and alcohol consumption.

Model 3: Model 2 + low-density lipoprotein cholesterol, systolic blood pressure, fasting glucose, smoking, physical activity, waist-to-hip ratio, high sensitivity C-reactive protein, albumin-to-creatinine ratio, and use of antihypertensive medications.

a A negative coefficient means a steeper decline; it was calculated using linear mixed model with random intercept and slope.

b Low HDL-C, ≤1.0 mmol/l (≤40 mg/dl); intermediate HDL-C, 1.1–1.6 mmol/l (41–61 mg/dl); high HDL-C, >1.6 mmol/l (>61 mg/dl).

**Table 3:** Association between baseline HDL-C levels and rapid GFR decline (GFR change rate < –3.0 ml/min per 1.73 m^2^ per year)

	Model 1			Model 2			Model 3			Model 4		
	OR	95% CI	*P* value	OR	95% CI	*P* value	OR	95% CI	*P* value	OR	95% CI	*P* value
HDL-C, per doubling (log2)	1.32	(0.91, 2.33)	0.27	1.98	(1.10, 3.58)	0.02	2.62	(1.38, 4.97)	0.00	2.70	(1.39, 5.22)	0.00
Low HDL-C^a^	Ref			Ref			Ref			Ref		
Intermediate HDL-C	1.06	(0.57, 1.98)	0.86	1.33	(0.67, 2.65)	0.41	1.48	(0.70, 3.13)	0.30	1.56	(0.72, 3.38)	0.26
High HDL-C	1.44	(0.75, 2.78)	0.28	2.21	(1.02, 4.79)	0.05	2.76	(1.20, 6.35)	0.02	2.97	(1.25, 7.07)	0.01

GFR, glomerular filtration rate; HDL-C, high-density lipoprotein cholesterol.

Model 1: Adjusted for sex and age.

Model 2: Age, sex, body mass index, triglycerides, use of lipid-lowering drugs, and alcohol consumption.

Model 3: Model 2 + low-density lipoprotein cholesterol, systolic blood pressure, fasting glucose, smoking, physical activity, waist-to-hip ratio, high sensitivity C-reactive protein, albumin-to-creatinine ratio, and use of antihypertensive medications.

Model 4: Model 3 + baseline GFR.

a Low HDL-C, ≤1.0 mmol/l (≤ 40 mg/dl); intermediate HDL-C, 1.1–1.6 mmol/l (41–61 mg/dl); high HDL-C, >1.6 mmol/l (>61 mg/dl).

**Figure 3:**
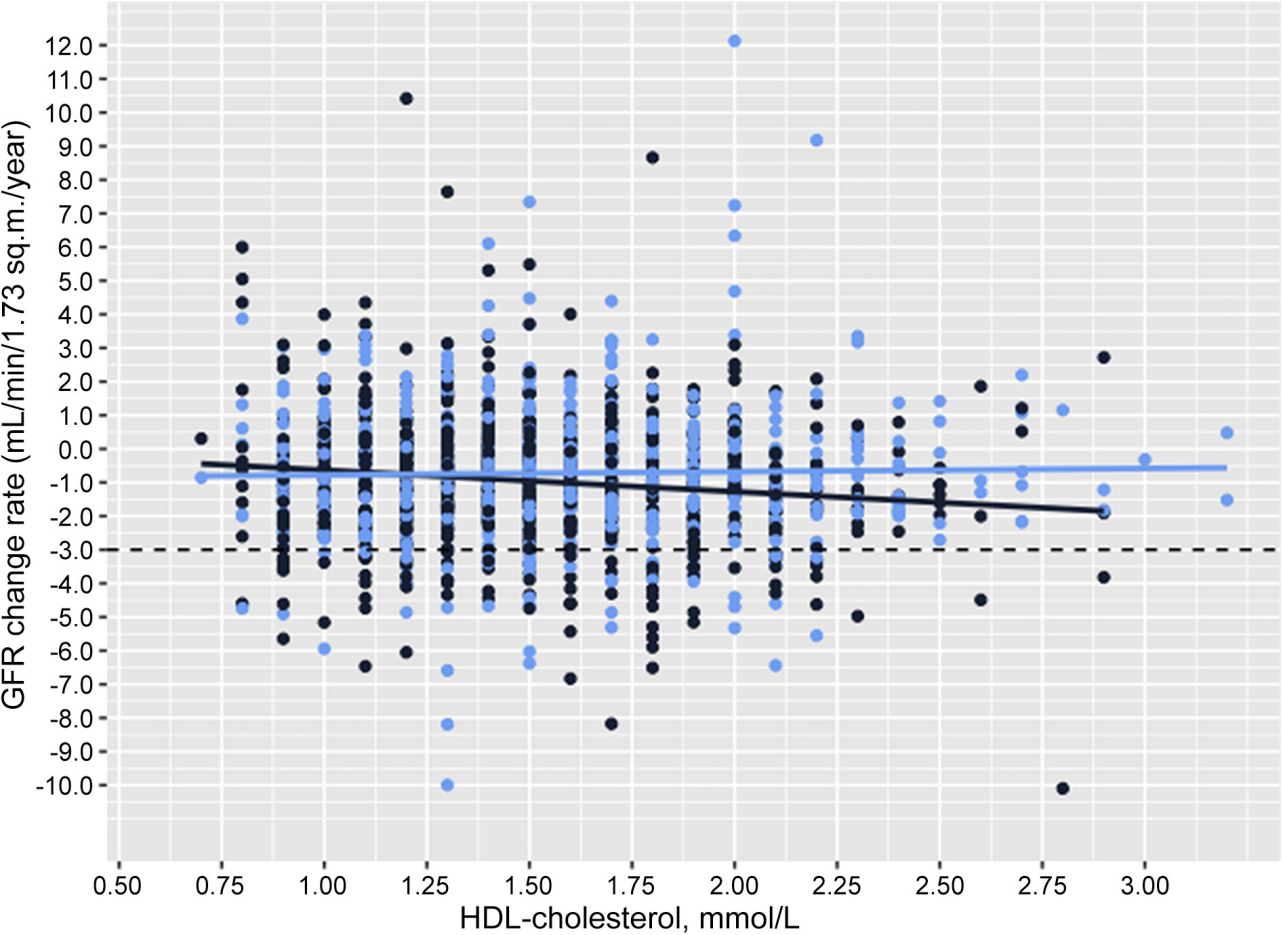
The association of HDL-C levels with annual GFR decline rates by physical active and inactive participants. Physical active persons are shown in light-blue dots and inactive persons in dark blue. (Unadjusted beta coefficient per doubling of HDL-C level for active versus inactive persons: -0.7 [95% CI –1.0, –0.3] ml/min per 1.72 m^2^ per year vs. 0.1 [95% CI –0.3, 0.5] ml/min per 1.72 m^2^ per year. *P* value for interaction < 0.01.) GFR, glomerular filtration rate; HDL-C, high-density lipoprotein cholesterol.

The associations with HDL-C were modified by physical activity for both the mean GFR decline rate (mixed linear regression) and odds ratio for rapid decline (*P* value for interaction < 0.01 and 0.04); the results stratified by physical activity are shown in Table 4. The association of HDL-C levels with GFR change rates (GFR_follow-up_ – GFR_baseline_ / observation time) were also calculated using linear regression; the results were essentially the same as in the mixed model analyses and are shown by physical activity group in Supplementary Table S2 and Figure 3.

**Table 4:** Association between baseline HDL-C levels and GFR decline by physical activity^a^

Annual GFR decline rate (*n* = 1623)^b^	Model 1			Model 2			Model 3		
	GFR, ml/min per 1.73 m^2^ per year	95% CI	*P* value	GFR, ml/min per 1.73 m^2^ per year	95% CI	*P* value	GFR, ml/min per 1.73 m^2^ per year	95% CI	*P* value
Inactive^c^ (*n* = 918); HDL-C, per doubling (log2)	–0.63	(–1.02, –0.24)	0.001	–0.82	(–1.30, –0.34)	0.001	–1.04	(–1.52, –0.55)	<0.001^d^
Active^c^ (*n* = 705); HDL-C, per doubling (log2)	0.17	(–0.25, 0.59)	0.44	–0.27	(–0.76, 0.21)	0.27	–0.29	(–0.80, 0.22)	0.26
Rapid GFR decline (*n* = 1321)^b^(GFR loss > 3 ml/min per 1.73 m^2^ per year)									
Inactive^c^ (*n* = 729); HDL-C, per doubling (log2)	2.02	(1.07, 3.82)	0.03	3.51	(1.55, 7.92)	<0.01	5.98	(2.33, 15.34)	<0.001^e^
Active^c^ (*n* = 592); HDL-C, per doubling (log2)	0.77	(0.35, 1.69)	0.51	0.94	(0.38, 2.31)	0.89	1.09	(0.41, 2.94)	0.86

GFR, glomerular filtration rate; HDL-C, high-density lipoprotein cholesterol.

Model 1: Adjusted for sex and age. Model 2: Model 1 + body mass index, low-density lipoprotein cholesterol (LDL-C), triglycerides, use of lipid-lowering drugs, and alcohol use.

Model 3: Model 2 + LDL-C, systolic blood pressure, fasting glucose, smoking, physical activity, waist-to-hip ratio, high-sensitivity C-reactive protein, albumin-to-creatinine ratio, and use of antihypertensive medications. For the rapid GFR decline outcome, we also included baseline GFR in model 3.

a Based on self-reported frequency and intensity of leisure-time physical activity (PA) as previously reported.^33^

b All participants were included regardless of number of GFR measurements, because linear mixed regression allows for missing observations at ≥1 time points. Only those with ≥2 GFR measurements were included in the logistic regression of rapid GFR decline. There were 4 missing values for PA (3 missing for PA for rapid GFR decline).

c Active (*>*1-h hard physical activity a week [becoming breathless or sweaty, or exhausted] and/or *>*3-h light activity [without becoming breathless or sweaty]) or inactive (all others).

d *P* value for interaction with physical activity < 0.01.

e *P* value for interaction with physical activity = 0.04.

No statistically significant interactions were found for age, sex, or hs-CRP on the association between HDL-C and odds ratio of rapid GFR decline assessed by logistic regression. However, the association of HDL-C with the mean GFR decline calculated using linear mixed model was stronger for men than for women and stronger for subjects with higher hs-CRP levels (*P* value for interaction ranged from 0.02 to 0.06 in model 1–3 for sex and from 0.01 to 0.02 for hs-CRP) (Supplementary Tables S3 and S4).

Twenty-six subjects developed stage 3 incident CKD, defined as new-onset mGFR <60 ml/min per 1.73 m^2^ at follow-up. The OR per doubling of HDL-C for incident CKD was 4.6 (95% CI 1.11, 19.2; *P* = 0.04) in the fully adjusted model (Supplementary Table S5).

There were no statistically significant nonlinear associations between HDL-C and GFR decline or the risk of rapid decline.

### Sensitivity Analyses

Thirty-four participants had a measured GFR <60 ml/min per 1.73 m^2^ and 42 had hs-CRP >20 mg/l at baseline. We excluded these participants to avoid possible bias due to a transient reduction in the GFR at baseline affecting predominantly those with low HDL-C levels. The results were comparable to those in the main analysis (Supplementary Tables S6 and S7).

To test whether a phase of hyperfiltration (increasing GFR from baseline to follow-up) in subjects with low HDL-C could have influenced our results, we excluded 138 persons with incident prediabetes (fasting glucose level of 6.1–7.0 mmol/l or an HbA_1c_ level of 6.0% to <6.5%) and 38 persons with incident diabetes (fasting glucose level of >7.0 mmol/l or an HbA_1c_ level of ≥6.5%) at follow-up. The results remained almost identical (Supplementary Table S8).

We repeated the logistic regression analyses using a different definition of rapid GFR decline, defined as the 10% steepest GFR slopes calculated using an adjusted linear mixed model.^40^^,^^41^ The association of HDL-C with this outcome was similar (Supplementary Table S9).

The results were also similar using sex-specific quartiles of HDL-C and another predefined categorization of physical activity (Supplementary Tables S10–S12).^34^

Finally, we repeated the analyses using the eGFR on the basis of the creatinine and/or cystatin C level (eGFRcrea, eGFRcys, and eGFRcreacys) as a dependent variable. The HDL-C levels were not associated with the mean GFR decline or risk of rapid eGFR decline using eGFRcys, but a similar tendency to the results using the measured GFR was found for eGFRcrea and eGFRcreacys, including a significant interaction between a rapid decline and physical activity using eGFRcreacys (Supplementary Tables S13–15).

## Discussion

In middle-aged subjects from the general population without pre-existing diabetes, CVD, or CKD, we found that higher HDL-C levels were independently associated with a steeper GFR decline and an increased risk of rapid GFR decline during a median of 5.6 years of follow-up.

Previous epidemiologic studies of HDL-C and the risk of kidney disease, including 3 Mendelian Randomization studies, reported inconsistent results.^6^, ^7^, ^8^^,^^42^ However, 5 population-based studies reported an association between low HDL-C levels and steeper rates of eGFR decline or a higher risk of incident CKD.^19^^,^^43^, ^44^, ^45^, ^46^

All these studies used estimates of GFR, and some studies included persons with diabetes or CKD. In most studies, the populations were not representative of the general population, and in several studies they did not adjust for relevant confounders.^19^^,^^43^, ^44^, ^45^, ^46^ Hypertriglyceridemia and abdominal obesity, in particular, correlate with lower HDL-C levels and have been linked to GFR decline and incident CKD in the general population.^39^

In the largest study of HDL-C levels and renal outcomes, consisting of 1,943,682 male veterans, the authors reported a U-shaped association of HDL-C with eGFR decline and end-stage kidney disease.^19^ In the current study, we did not observe any nonlinear associations between HDL-C and the outcomes, possibly because few had low HDL-C levels, as we included relatively healthy subjects. Conversely, in the US Veteran study, 31% of the subjects had diabetes, 33% had CVD, 40% had obesity, and 52% used statins at baseline. The increased risk associated with higher HDL-C levels in the US Veteran study started at approximately 55 mg/dl (1.42 mmol/l), corresponding to the median HDL-C level in our study.

HDL-C is traditionally regarded as “good” cholesterol, and the association of higher HDL-C with the loss of the GFR may seem counterintuitive. Several hypotheses can be raised as explanations for our findings.

Persons with high HDL-C levels may suffer from other conditions that can influence the GFR decline rate, such as inflammation or alcohol abuse.^29^^,^^47^ However, the inclusion of hs-CRP, cardiovascular risk factors and alcohol consumption as covariates strengthened rather than attenuated the association.

Experimental evidence suggests that high levels of HDL-C per se, or higher levels of dysfunctional HDL-C, contribute to endothelial dysfunction and vascular disease.^9^^,^^11^^,^^48^ Although very low HDL-C levels may enhance endothelial dysfunction, it has been demonstrated that moderate to high HDL-C levels (1.0–2.1 mmol/l [40–80 mg/dl]) obtained from healthy subjects paradoxically enhanced the senescence of human endothelial progenitor cells and related angiogenesis.^48^

We did not measure HDL-C dysfunction in the current study; however, previous studies have shown that HDL-C from healthy nonobese elderly persons contains higher levels of glycosylated apoA-1 and exhibits a lower antioxidative ability than does HDL-C from younger persons.^13^ The treatment of human dermal fibroblasts and macrophages with HDL-C isolated from elderly subjects (mean age 71 ± 4 years) increased cellular senescence and foam cell formation, whereas treatment with HDL-C from young adults suppressed senescence and atherosclerosis.^11^

Smaller modified HDL-C particles and HDL components, such as ApoA1, may interact with several renal cell classes, as they are filtered in the glomeruli and reabsorbed in the proximal tubuli.^16^ Indeed, oxidized HDL-C enhances the production of reactive oxygen species and upregulates the expression of proinflammatory factors in human proximal tubule epithelial cells in a dose-dependent manner.^15^

Associations of higher HDL-C levels with the GFR change rate and risk of rapid GFR decline were found in subjects who reported performing little or no physical activity, and the association with the GFR change rate was significant for men and subjects with higher hs-CRP levels only. Although the results of subgroup analyses should be interpreted with caution, we speculate that the effect modifications of hs-CRP, physical activity, and sex may be explained by altered HDL-C functionality. Experimental studies showed that low-grade inflammation modulates the composition and function of human HDL-C, leading to the loss of endothelial protective properties.^9^^,^^12^

Physical activity, on the other hand, and particularly aerobic exercise, has been shown to reduce low-grade inflammation and to improve the antioxidant and anti-inflammatory effects of HDL-C.^24^^,^^25^ Whether HDL-C in part mediates a possible deleterious effect of inflammation on GFR loss or vice versa and whether this can be prevented by physical activity should be addressed in future studies. A study of statin treatment in subjects with high levels of dysfunctional HDL-C may also be warranted, as the inflammatory properties of dysfunctional HDL-C may be improved by simvastatin.^9^^,^^10^

We observed a stronger association of HDL-C with the mean GFR decline rate in men than in women. Several sex-specific differences have been reported in the etiology and epidemiology of CKD, but the underlying mechanisms are unclear. Sex differences in vascular function, HDL oxidation leading to dysfunctional HDL-C, and inflammation in the kidneys may potentially influence the association of HDL-C with GFR decline.^49^

The main strength of the current study is the GFR measurements in a well-described general population cohort. Our results were robust when both linear mixed and logistic regression models were used. Some limitations should be mentioned. We investigated middle-aged persons mainly of North European ancestry; thus, our results cannot necessarily be generalized to other age groups or ethnicities. We did not include markers of dysfunctional HDL-C or objective measures of physical activity. The observation time was limited to 5.6 years, and the vast majority of subjects had 1 follow-up GFR only.

We conclude that higher HDL-C levels were associated with a steeper GFR decline rate and increased the risk of rapid GFR decline in middle-aged subjects without diabetes and pre-existing CKD. Effect modifications indicated stronger associations of HDL-C with GFR loss in physically inactive persons, in those with higher hs-CRP levels and in men. This complex association of HDL-C with GFR loss should be addressed in future studies.

## Disclosure

BOE, TM, and VTS report an unrestricted grant from Boehringer-Ingelheim to the RENIS-FU study. The funding source had no role in the design and conduct of the study. ITE, JVN, MDS, RR, and TJ have no relevant financial relationships to disclose.

## Supplementary Material

Supplementary File (PDF)

**Table S1.** Study population at baseline by sex-specific quartiles of HDL cholesterol.

**Table S2.** Association of HDL-C levels with GFR decline calculated by linear regression.

**Table S3.** Association between baseline HDL-C levels and annual GFR change rates by sex.

**Table S4.** Association between baseline HDL-C levels and GFR change rates by hs-CRP.

**Table S5.** Association between baseline HDL-C levels and incident CKD stage 3.

**Table S6.** Association between baseline HDL-C levels and GFR change rates in participants with baseline GFR >60 ml/min per 1.73 m^2^.

**Table S7.** Association between baseline HDL-C levels and GFR change rates in participants with hs-CRP <20 mg/l.

**Table S8.** Association of baseline HDL-C levels with GFR decline when subjects with incident diabetes or prediabetes were excluded.

**Table S9.** Association between baseline HDL-C levels and rapid GFR decline defined as 10% steepest decline rate.

**Table S10.** Association between baseline HDL-C levels and annual GFR change rates.

**Table S11.** Association between baseline HDL-C levels and rapid GFR decline.

**Table S12.** Association between baseline HDL-C levels and GFR decline by an alternative physical activity category.

**Table S13.** Association of baseline HDL-C levels with eGFR decline using the creatinine-based CKD-EPI equation.

**Table S14.** Association of baseline HDL-C levels with eGFR decline using the cystatin-C–based CKD-EPI equation.

**Table S15.** Association of baseline HDL-C levels with eGFR decline using the combined creatinine- and cystatin-based CKD-EPI equation.
