# Supplementary material for: Association of High-Density Lipoprotein Cholesterol With GFR Decline in a General Nondiabetic Population
Source: Kidney Int Rep. 2021 May 19;6(8):2084–94. doi: 10.1016/j.ekir.2021.05.007 (PMC8343778; doi:10.1016/j.ekir.2021.05.007)
Supplement: Supplementary File (PDF) [file mmc1.pdf]

Supplemental material Table of Contents

Table S1. Study population at baseline by sex-specific quartiles of HDL-Cholesterol.

Table S2. Association of HDL-C levels with GFR decline calculated by linear regression.

Table S3. Association between baseline HDL-C levels and annual GFR change rates by sex

Table S4. Association between baseline HDL-C levels and GFR change rates by hs-CRP

Table S5. Association between baseline HDL-C levels and incident CKD stage 3

Table S6. Association between baseline HDL-C levels and GFR change rates in participants with baseline GFR>60 ml/min/1.73 m^2

Table S7. Association between baseline HDL-C levels and GFR change rates in participants with hs-CRP < 20 mg/L

Table S8. Association of baseline HDL-C levels with GFR decline when subjects with incident diabetes or prediabetes were excluded.

Table S9. Association between baseline HDL-C levels and rapid GFR decline defined as 10% steepest decline rate.

Table S10. Association between baseline HDL-C levels and annual GFR change rates.

Table S11. Association between baseline HDL-C levels and rapid GFR decline.

Table S12. Association between baseline HDL-C levels and GFR decline by an alternative physical activity category.

Table S13. Association of baseline HDL-C levels with eGFR decline using the creatinine-based CKD-EPI equation.

Table S14. Association of baseline HDL-C levels with eGFR decline using the cystatin-C based CKD-EPI equation.

Table S15. Association of baseline HDL-C levels with eGFR decline using the combined creatinine- and cystatin-based CKD-EPI equation.

Table S1. Study population at baseline by sex-specific quartiles of HDL-Cholesterol

Characteristics	Quartile of HDL-C (interquartile range)				p-value (linear trend)
	Quartile 1	Quartile 2	Quartile 3	Quartile 4	
women	(0.4-1.3 mmol/L)	(1.4-1.5 mmol/L)	(1.6-1.8 mmol/L)	(1.9-3.2 mmol/L)	
men	(0.7-1.0 mmol/L)	(1.1-1.2 mmol/L)	(1.3-1.5 mmol/L)	(1.6-2.9 mmol/L)	
Participants, n	320[a]	358[a]	470[a]	479[a]	
Women, n	180[a] (56%)	174[a] (49%)	210[a] (45%)	262[a] (55%)	
Age, yr	58.0 (54.0-61.3)	58.9 (54.2-61.3)	58.7 (55.3-61.4)	59.0 (54.9-61.6)	0.006
Body mass index, kg/m²	28.5 (26.3-31.1)	27.6 (25.5-30.5)	27.0 (24.8-29.6)	25.0 (23.0-27.6)	<0.001
Waist-hip ratio	0.93 (0.89-0.98)	0.92 (0.87-0.97)	0.91 (0.87-0.97)	0.88 (0.83-0.93)	<0.001
Systolic blood pressure, mmHg	127 (118-141)	129 (117-142)	130 (117-140)	127 (116-139)	0.08
Diastolic blood pressure, mmHg	83 (77-89)	84 (77-90)	84 (78-91)	82 (75-88)	0.06
Blood preassure medication, n	75 (23%)	88 (25%)	74 (16%)	62 (13%)	<0.001
Fasting blood glucose, mmol/L	5.4 (5.1-5.8)	5.5 (5.0-5.6)	5.3 (5.0-5.7)	5.2 (4.9-5.6)	<0.001
Total cholesterol, mmol/L	5.6 (4.8-6.3)	5.6 (4.9-6.1)	5.6 (5.0-6.3)	5.6 (5.2-6.3)	0.01
LDL-C, mmol/L	3.8 (3.3-4.4)	3.8 (3.2-4.3)	3.7 (3.1-4.2)	3.4 (2.8-3.9)	<0.001
HDL-C, mmol/L	1.0 (0.9-1.2)	1.2 (1.2-1.5)	1.5 (1.4-1.7)	2.0 (1.8-2.2)	
Triglycerides, mmol/L	1.6 (1.1-2.1)	1.2 (0.9-1.6)	1.0 (0.8-1.3)	0.8 (0.6-1.0)	<0.001
Lipid lowering medication, n	23 (7%)	23 (6%)	32 (7%)	29 (6%)	0.6
High-sensitivity CRP, mg/L	1.71 (1.0-3.3)	1.39 (0.73-2.55)	1.11 (0.63-2.18)	0.92 (0.50-1.63)	<0.001
Daily smoker, n					<0.001
never	95 (30%)	89 (25%)	157 (33%)	163 (34%)	
yes, previously	132 (41%)	183 (51%)	232 (49%)	224 (47%)	
yes, currently	90 (28%)	85 (24%)	81 (17%)	88 (18%)	
Alcohol use, n					<0.001
Once a month or less	141 (44%)	98 (27%)	126 (27%)	103 (21%)	
2-4 times a month	135 (42%)	164 (46%)	222 (47%)	196 (41%)	
2 times a week or more	44 (14%)	96 (27%)	122 (26%)	180 (38%)	
Physical activity[b], n					
>1-h high-intensity and/or >3-h low-intensity per week	125 (39%)	142 (40%)	196 (42%)	242 (51%)	0.001
Urinary ACR, mg/mmol	0.31 (0.10-0.59)	0.25 (0.10-0.53)	0.15 (0.10-0.49)	0.23 (0.10-0.56)	0.004[c]
mGFRiohexol, mL/min/1.73 m²	95.5 (83.6-104.5)	92.8 (84.0-101.6)	94.2 (86.1-104.5)	93.3 (85.4-102.9)	0.02

Data is presented as means (SD) and median (IQ range) for continous variables and n (%) for dichomous variables .

[a]The number of participants in the different quartiles of HDL are not equal because only one decimal is given from the laboratory (tied values).

[b]Based on self-reported leisure-time physical exercise: Active [>1-h hard physical activity a week (becoming breathless or sweaty, or exhausted) and/or >3-h light activity (without becoming breathless or sweaty)] or inactive (all others)

Missing values: 7 for alcohol consumption, 8 for smoking, 18 for hs-CRP, 39 for wasit-hip ratio, 5 for ACR and 4 physical activity.

[c]P = 0.005 for quadratic trend between HDL-C and urinary ACR (logACR)

Table S2. Association between baseline HDL-C levels and GFR decline calculated using linear regression, grouped by physical activity [a]

	Model 1			Model 2			Model 3		
**Annual GFR decline rate[b]**	mL/min /1.73 m^2 per year	95% CI	P	mL/min /1.73 m^2 per year	95% CI	P	mL/min /1.73 m^2 per year	95% CI	P
Inactive[c] (N=729)									
HDL-C, per doubling (log2)	-0.65	(-1.05 to -0.25)	0.001	-0.91	(-1.41 to -0.42)	<0.001	-1.07	(-1.53 to -0.59)	<0.001[d]
Active[c] (N=592)									
HDL-C, per doubling (log2)	0.15	(-0.29 to 0.60)	0.50	-0.21	(-0.72 to 0.29)	0.41	-0.12	(-0.59 to 0.35)	0.62

[a]Based on self-reported frequency and intensity of leisure-time physical activity (PA) as previously reported (ref 33).

[b]Calculated as $GFR_{follow-up} – GFR_{baseline}$ /observation time in years. Only those with ≥ 2 GFR measurements were included in the this analysis.

[c]Active [>1-h hard physical activity a week (becoming breathless or sweaty, or exhausted) and/or >3-h light activity (without becoming breathless or sweaty)] or inactive (all others). [d]P-value for interaction with physical activity < 0.01.

Model 1: Adjusted for sex and age. Model 2: Model 1 + body mass index (BMI), triglycerides, use of lipid-lowering drugs and alcohol use.

Model 3: Model 2 + low density lipoprotein (LDL), systolic BP, fasting glucose, smoking, physical activity, waist-to-hip ratio, hs-CRP, albumin-to-creatinine ratio (ACR), baseline GFR and use of antihypertensive medications.

Table S3. Association between baseline HDL-C levels and annual GFR change rates[a] by sex

	Model 1			Model 2			Model 3		
	mL/min /1.73 m² per year	(95% CI)	p value	mL/min /1.73 m² per year	(95% CI)	p value	mL/min /1.73 m² per year	(95% CI)	p value
**ALL 1627**									
HDL-C, per doubling (log2)	-0.22	(-0.51 to 0.06)	0.13[b]	-0.53	(-0.87 to -0.18)	0.003[b]	-0.64	(-0.99 to -0.29)	<0.001[b]
Low HDL-C (≤1.0 mmol/L)	Ref			Ref			Ref		
Intermediate HDL-C (1.1-1.6 mmol/L)	-0.11	(-0.46 to 0.23)	0.53	-0.32	(-0.70 to 0.06)	0.10	-0.29	(-0.67 to 0.10)	0.15
High HDL-C (>1.6 mmol/L)	-0.20	(-0.57 to 0.18)	0.30	-0.51	(-0.95 to -0.08)	0.02	-0.53	(-0.97 to -0.08)	0.02
**Females (N=826)**									
HDL-C, per doubling (log2)	0.09	(-0.32 to 0.50)	0.67	-0.28	(-0.78 to 0.21)	0.26	-0.38	(-0.88 to 0.13)	0.14
Low HDL-C (≤1.0 mmol/L)	Ref			Ref			Ref		
Intermediate HDL-C (1.1-1.6 mmol/L)	0.08	(-0.60 to 0.76)	0.81	-0.25	(-0.96 to 0.46)	0.48	-0.28	(-1.01 to 0.45)	0.46
High HDL-C (>1.6 mmol/L)	0.15	(-0.52 to 0.83)	0.66	-0.34	(-1.09 to 0.42)	0.38	-0.37	(-1.14 to 0.41)	0.36
**Males (N=801)**									
HDL-C, per doubling (log2)	-0.51	(-0.91 to -0.12)	0.01	-0.78	(-1.26 to -0.30)	0.002	-0.92	(-1.41 to -0.43)	<0.001
Low HDL-C (≤1.0 mmol/L)	Ref			Ref			Ref		
Intermediate HDL-C (1.1-1.6 mmol/L)	-0.15	(-0.55 to 0.25)	0.47	-0.28	(-0.72 to 0.17)	0.23	-0.25	(-0.71 to 0.20)	0.28
High HDL-C (>1.6 mmol/L)	-0.49	(-0.98 to -0.01)	0.04	-0.70	(-1.27 to -0.14)	0.01	-0.81	(-1.38 to -0.23)	0.01

[a]A negative coefficient means a steeper decline. Calculated using linear mixted model with random intercept and slope.

[b]P-value for interaction was 0.036, 0.020 and 0.055 in model 1-3.

Model 1: Adjusted age

Model 2: Model 1 + body mass index (BMI), triglycerides, use of lipid-lowering drugs and alcohol consumption

Model 3: Model 2 + low density lipoprotein (LDL), systolic blood pressure, fasting glucose, smoking, physical activity, waist-to-hip ratio (WHR), hs-CRP, albumin-to-creatinine ratio (ACR) and use of antihypertensive medications.

Table S4. Association between baseline HDL-C levels and annual GFR change rates[a] by high-sensitivity CRP

	**Model 1**				**Model 2**				**Model 3**			
	mL/min /1.73 m^2 per year	95% CI		p value	mL/min /1.73 m^2 per year	95% CI		p value	mL/min /1.73 m^2 per year	95% CI		p value
**Tertile 1** (CRP: 0.10-0.79 mg/L)												
HDL-C, per doubling (log2)	-0.13	(-0.64	0.38)	0.6[b]	-0.32	(-0.95	0.29)	0.3[b]	-0.34	(-0.99	0.31)	0.3[b]
**Tirtile 2** (CRP: 0.80- 1.75 mg/L)												
HDL-C, per doubling (log2)	-0.40	(-0.87	0.06)	0.09	-0.76	(-1.30	-0.22)	<0.01	-0.93	(-1.49	-0.37)	0.001
**Tirtile 3** (CRP: 1.76-174 mg/L)												
HDL-C, per doubling (log2)	-0.39	(-0.96	0.17)	0.17	-0.53	(-1.18	0.11)	0.11	-0.50	(-1.15	0.15)	0.13

[a]A negative coefficient means a steeper decline. Calculated using linear mixted model with random intercept and slope.

[b]P-value for interaction between HDL-C and hs-CRP as a continous variable was 0.016, 0.012 and 0.019 in model 1-3.

Model 1: Adjusted for sex and age

Model 2: Model 1 + body mass index (BMI), triglycerides, use of lipid-lowering drugs and alcohol consumption

Model 3: Model 2 + low density lipoprotein (LDL), systolic blood pressure, fasting glucose, smoking, physical activity, waist-to-hip ratio (WHR), albumin-to-creatinine ratio (ACR) and use of antihypertensive medications.

Table S5. Association between baseline HDL-C levels and incident CKD stage 3 (defined as new onset GFR<60 ml/min/1.73 m^2)

	Model 1			Model 2			Model 3			Model 4		
	OR	95% CI	p-value	OR	95% CI	p-value	OR	95% CI	p-value	OR	95% CI	p-value
HDL-C, per doubling (log2)	1.28	(0.45 - 3.69)	0.64	2.37	(0.68 - 8.22)	0.18	4.68	(1.13 - 19.50)	0.03	4.62	(1.11 - 19.24)	0.04

Model 1: Adjusted for sex and age

Model 2: Sex, age, BMI, triglycerides, use of lipid-lowering drugs and alcohol consumption.

Model 3: Model 2 + low density lipoprotein (LDL), systolic blood pressure, fasting glucose, smoking, physical activity, WHR, hs-CRP, albumin-to-creatinine ratio (ACR) and use of antihypertensive medications.

Model 4: Model 3 + baseline GFR

Table S6. Association between baseline HDL-C levels and annual GFR change rates[a] in participants with baseline GFR>60 ml/min/1.73 m^2

	Model 1			Model 2			Model 3		
	mL/min /1.73 m^2 per year	95% CI	p value	mL/min /1.73 m^2 per year	95% CI	p value	mL/min /1.73 m^2 per year	95% CI	p value
HDL-C, per doubling (log2)	-0.28	(-0.55 to -0.01)	0.04	-0.60	(-0.92 to -0.27)	<0.001	-0.72	(-1.05 to -0.38)	<0.001

[a]A negative coefficient means a steeper decline. Calculated using linear mixted model with random intercept and slope.

Model 1: Adjusted for sex and age

Model 2: Model 1 + body mass index (BMI), triglycerides, use of lipid-lowering drugs and alcohol consumption

Model 3: Model 2 + low density lipoprotein (LDL), systolic blood pressure, fasting glucose, smoking, physical activity, waist-to-hip ratio (WHR), hs-CRP, albumin-to-creatinine ratio (ACR) and use of antihypertensive medications.

Table S7. Association between baseline HDL-C levels and annual GFR change rates[a] in participants with hs-CRP < 20 mg/L

	Model 1			Model 2			Model 3		
	mL/min /1.73 m^2 per year	95% CI	p value	mL/min /1.73 m^2 per year	95% CI	p value	mL/min /1.73 m^2 per year	95% CI	p value
HDL-C, per doubling (log2)	-0.20	(-0.49 to 0.09)	0.17	-0.53	(-0.87 to -0.19)	<0.01	-0.60	(-0.95 to -0.25)	0.001

[a]A negative coefficient means a steeper decline. Calculated using linear mixted model with random intercept and slope.

Model 1: Adjusted for sex and age

Model 2: Model 1 + body mass index (BMI), triglycerides, use of lipid-lowering drugs and alcohol consumption

Model 3: Model 2 + low density lipoprotein (LDL), systolic blood pressure, fasting glucose, smoking, physical activity, waist-to-hip ratio (WHR), hs-CRP, albumin-to-creatinine ratio (ACR) and use of antihypertensive medications.

Table S8. Association of baseline HDL-C levels with GFR decline when subjects with incident diabetes or prediabetes were excluded.

Annual GFR decline rate	Model 1			Model 2			Model 3		
	mL/min /1.73 m^2 per year	95% CI	p value	mL/min /1.73 m^2 per year	95% CI	p value	mL/min /1.73 m^2 per year	95% CI	p value
HDL-C, per doubling (log2)	-0.23	(-0.55 to 0.08)	0.15	-0.56	(-0.94 to -0.19)	0.003	-0.68	(-1.06 to -0.29)	0.001
Low HDL-C	Ref			Ref			Ref		
Intermediate HDL-C	-0.04	(-0.43 to 0.36)	0.85	-0.29	(-0.72 to 0.14)	0.19	-0.37	(-0.82 to 0.07)	0.10
High HDL-C	-0.16	(-0.58 to 0.26)	0.46	-0.52	(-1.01 to -0.03)	0.04	-0.63	(-1.14 to -0.13)	0.01
**Rapid GFR decline**	**Model 1**			**Model 2**			**Model 3**		
(GFR loss > 3 ml/min/1.73 m^2/year)	OR	95% CI	p value	OR	95% CI	p value	OR	95% CI	p value
HDL-C, per doubling (log2)	1.56	(0.90 to 2.70)	0.11	2.19	(1.13 to 4.22)	0.02	2.87	(1.39 to 5.93)	0.004
Low HDL-C	Ref			Ref			Ref		
Intermediate HDL-C	1.27	(0.58 to 2.77)	0.55	1.67	(0.70 to 3.96)	0.25	2.30	(0.83 to 6.40)	0.11
High HDL-C	1.83	(0.82 to 4.09)	0.14	2.81	(1.09 to 7.24)	0.03	4.22	(1.39 to 12.77)	0.01

Model 1: Adjusted for sex and age.

Model 2: Model 1 + BMI, low density lipoprotein (LDL), triglycerides, use of lipid-lowering drugs and alcohol consumption.

Model 3: Model 2 + systolic blood pressure, fasting glucose, smoking, physical activity, WHR, hs-CRP, albumin-to-creatinine ratio (ACR) and use of antihypertensive medications. In the logistic regression model 3 for rapid GFR decline we also included baseline GFR.

Table S9. Association between baseline HDL-C levels and rapid GFR decline defined as 10% steepest decline rate [a]

	Model 1			Model 2			Model 3			Model 4		
	OR	95% CI	p-value	OR	95% CI	p-value	OR	95% CI	p-value	OR	95% CI	p-value
HDL-C, per doubling (log2)	1.09	(0.66 - 1.80)	0.72	2.14	(1.18 - 3.91)	0.01	2.77	(1.45 - 5.29)	0.00	2.93	(1.50 - 5.75)	0.00
Low HDL-C	Ref			Ref			Ref			Ref		
Intermediate HDL-C	0.90	(0.49 - 1.66)	0.73	1.42	(0.72 - 2.82)	0.31	1.43	(0.69 - 2.96)	0.34	1.54	(0.72 - 3.32)	0.27
High HDL-C	1.13	(0.59 - 2.16)	0.71	2.39	(1.10 - 5.21)	0.03	2.81	(1.24 - 6.36)	0.01	3.16	(1.33 - 7.47)	0.01

[a]Defined as 10% steepest GFR decline, calculated using an multivariable adjusted linear mixted model; GFR change rate < - 1.9 mL/min/1.73 m^2/year.

Model 1: Adjusted for sex and age.

Model 2: Model 1 + BMI, triglycerides, use of lipid-lowering drugs and alcohol consumption.

Model 3: Model 2 + low density lipoprotein (LDL), systolic blood pressure, fasting glucose, smoking, physical activity, WHR, hs-CRP, albumin-to-creatinine ratio (ACR) and use of antihypertensive medications.

Model 4: Model 3 + baseline GFR.

Table S10. Association between baseline HDL-C levels and annual GFR change rates

	Model 1			Model 2			Model 3		
	mL/min /1.73 m^2 per year[a]	(95% CI)	p value	mL/min /1.73 m^2 per year[a]	(95% CI)	p value	mL/min /1.73 m^2 per year[a]	(95% CI)	p value
Sex-specific quartiles									
HDL-C, 1st quartile	Ref			Ref			Ref		
HDL-C, 2nd quartile	0.18	(-0.14 to 0.50)	0.27	0.06	(-0.28 to 0.39)	0.74	0.02	(-0.31 to 0.36)	0.89
HDL-C, 3rd quartile	0.07	(-0.24 to 0.38)	0.65	-0.09	(-0.43 to 0.24)	0.59	-0.17	(-0.51 to 0.17)	0.32
HDL-C, 4th quartile	-0.08	(-0.38 to 0.23)	0.62	-0.30	(-0.66 to 0.05)	0.09	-0.41	(-0.77 to -0.05)	0.03

[a]A negative coefficient means a steeper decline. Calculated using linear mixted model with random intercept and slope.

Model 1: Adjusted for sex and age

Model 2: Model 1 + body mass index (BMI), triglycerides, use of lipid-lowering drugs and alcohol consumption

Model 3: Model 2 + low density lipoprotein (LDL), systolic BP, fasting glucose, smoking, physical activity, waist-to-hip ratio (WHR), high sensitivity C-reactive protein (hs-CRP), albumin-to-creatinine ratio (ACR) and use of antihypertensive medications.

Table S11. Association between baseline HDL-C levels and rapid GFR decline (GFR change rate < -3.0 mL/min/1.73 m^2/year)

	Model 1			Model 2			Model 3			Model 4		
	OR	95% CI	p-value	OR	95% CI	p-value	OR	95% CI	p-value	OR	95% CI	p-value
Sex-specific quartiles												
HDL-C, 1st quartile	Ref			Ref			Ref			Ref		
HDL-C, 2nd quartile	0.78	(0.43 - 1.40)	0.40	0.95	(0.51 - 1.76)	0.86	1.15	(0.58 - 2.26)	0.69	1.29	(0.65 - 2.58)	0.47
HDL-C, 3rd quartile	1.08	(0.64 - 1.83)	0.78	1.38	(0.76 - 2.51)	0.29	1.94	(1.01 - 3.73)	0.05	1.86	(0.96 - 3.62)	0.07
HDL-C, 4th quartile	1.10	(0.66 - 1.85)	0.71	1.63	(0.88 - 3.04)	0.12	2.29	(1.16 - 4.54)	0.02	2.33	(1.16 - 4.69)	0.02

Model 1: Adjusted for sex and age. Model 2: Age, sex, BMI, triglycerides, use of lipid-lowering drugs and alcohol consumption.
Model 3: Model 2 + low density lipoprotein (LDL), systolic BP, fasting glucose, smoking, physical activity, waist-to-hip ratio,
high sensitivity C-reactive protein (hs-CRP), albumin-to-creatinine ratio (ACR) and use of antihypertensive medications. Model 4: Model 3 + baseline GFR

Table S12. Association between baseline HDL-C levels and GFR decline by physical activity using a different categorization of physical activity [a]

Annual GFR decline rate[b]	Model 1			Model 2			Model 3		
	mL/min /1.73 m^2 per year	95% CI	p value	mL/min /1.73 m^2 per year	95% CI	p value	mL/min /1.73 m^2 per year	95% CI	p value
Never or low-intensity exercise[c]									
HDL-C, per doubling (log2) (N=704)	-0.74	(-1.19 to -0.28)	0.00	-0.92	(-1.48 to -0.37)	0.00	-1.15	(-1.71 to -0.59)	<0.001
Moderate-high intensity exercise[d]									
HDL-C, per doubling (log2) (N=863)	0.09	(-0.29 to 0.47)	0.64	-0.30	(-0.74 to 0.15)	0.19	-0.34	(-0.80 to 0.13)	0.154

Rapid GFR decline[b] (GFR loss > 3 ml/min/1.73 m2/year)	Model 1			Model 2			Model 3		
	OR	95% CI	p value	OR	95% CI	p value	OR	95% CI	p value
Never or low-intensity exercise[c]									
HDL-C, per doubling (log2) (N=566)	2.45	(1.14 to 5.27)	0.02	4.44	(1.69 to 11.66)	0.002	7.52	(2.41 to 23.46)	0.001
Moderate-high intensity exercise[d]									
HDL-C, per doubling (log2) (N=714)	0.85	(0.44 to 1.67)	0.64	1.07	(0.49 to 2.36)	0.87	1.31	(0.55 to 3.11)	0.55

[a]Based on self-reported frequency and intensity of leisure-time physical activity (PA) as previously reported (ref 34).

[b]All participants were included regardless of number of GFR measurements, because linear mixed regression allows for missing observations at ≥ 1 timepoints. 60 missing for PA.

Only those with a follow-up GFR were included in the logistic regression of rapid GFR decline, except for 44 with missing PA data.

[c]"Easy exercise; without becoming breathless or sweaty", [d]"moderate to hard exercise; becoming breathless and sweaty, or exhausted", ≥ once a week (34).

Model 1: Adjusted for sex and age.

Model 2: Model 1 + BMI, low density lipoprotein (LDL), triglycerides, use of lipid-lowering drugs and alcohol consumption.

Model 3: Model 2 + systolic blood pressure, fasting glucose, smoking, WHR, hs-CRP, albumin-to-creatinine ratio (ACR) and use of antihypertensive medications.

In the logistic regression model 3 for rapid GFR decline we also included baseline GFR.

Table S13. Association of baseline HDL-C levels with eGFR decline using the creatinine-based CKD-EPI equation.

	Model 1			Model 2			Model 3		
**Annual eGFR decline rate**	mL/min /1.73 m^2 per year	95% CI	p value	mL/min /1.73 m^2 per year	95% CI	p value	mL/min /1.73 m^2 per year	95% CI	p value
All (N=1627)[a]									
HDL-C, per doubling (log2)	-0.10	(-0.27 - 0.07)	0.25	-0.19	(-0.40 - 0.02)	0.07	-0.21	(-0.42 - 0.01)	0.06[c]
Inactive[b] (N=918)									
HDL-C, per doubling (log2)	-0.19	(-0.43 - 0.05)	0.11	-0.26	(-0.55 - 0.04)	0.09	-0.29	(-0.60 - 0.02)	0.06
Active[b] (N=705)									
HDL-C, per doubling (log2)	0.01	(-0.25 - 0.26)	0.94	-0.10	(-0.39 - 0.20)	0.52	-0.11	(-0.41 - 0.19)	0.48

**Rapid eGFR decline**	Model 1			Model 2			Model 3		
(GFR loss > 3 ml/min/1.73 m^2/year)	OR	95% CI	p value	OR	95% CI	p value	OR	95% CI	p value
All (N=1329)[a]									
HDL-C, per doubling (log2)	1.05	(0.57 - 1.93)	0.87	1.39	(0.67 - 2.90)	0.38	1.32	(0.61 - 2.85)	0.49[c]
Inactive[b] (N=731)									
HDL-C, per doubling (log2)	1.51	(0.65 - 3.53)	0.34	2.11	(0.73 - 6.14)	0.17	2.01	(0.60 - 6.66)	0.26
Active[b] (N=595)									
HDL-C, per doubling (log2)	0.69	(0.28 - 1.68)	0.41	0.88	(0.32 - 2.43)	0.81	0.79	(0.28 - 2.21)	0.65

[a]All participants were included regardless of number of eGFR measurements, because linear mixed regression allows for missing observations at ≥ 1 timepoints.
Only those with ≥ 2 eGFR measurements were included in the logistic regression of rapid GFR decline. 4 missing values for physical activity.
[b]Self-reported frequency and intensity of leisure-time physical exercise as previously reported (ref 33). Active [>1-h hard physical activity a week (becoming breathless or sweaty, or exhausted) and/or >3-h light activity (without becoming breathless or sweaty)] or inactive (all others).
[c]P-value for interaction with physical activity = 0.1. Model 1: Adjusted for sex and age. Model 2: Model 1 + BMI, low density lipoprotein (LDL), triglycerides, use of lipid-lowering drugs and alcohol consumption. Model 3: Model 2 + systolic blood pressure, fasting glucose, smoking, physical activity, waist to hip ratio, hs-CRP, albumin-to-creatinine ratio (ACR) and use of antihypertensive medications. In the logistic regression model 3 for rapid GFR decline we also included baseline GFR. Physical Activity was not included as a covariate in the stratified analyses.

Table S14. Association of baseline HDL-C levels with eGFR decline using the cystatin-C based CKD-EPI equation.

	Model 1			Model 2			Model 3		
**Annual eGFR decline rate**	mL/min /1.73 m^2 per year	95% CI	p value	mL/min /1.73 m^2 per year	95% CI	p value	mL/min /1.73 m^2 per year	95% CI	p value
All (N=1627)[a]									
HDL-C, per doubling (log2)	0.01	(-0.21 - 0.24)	0.914	0.06	(-0.21 - 0.33)	0.659	0.05	(-0.23 - 0.33)	0.75[c]
Inactive[b] (N=918)									
HDL-C, per doubling (log2)	-0.12	(-0.43 - 0.18)	0.431	-0.01	(-0.38 - 0.37)	0.967	-0.06	(-0.45 - 0.33)	0.77
Active[b] (N=705)									
HDL-C, per doubling (log2)	0.20	(-0.13 - 0.53)	0.23	0.16	(-0.22 - 0.55)	0.40	0.17	(-0.23 - 0.56)	0.40

**Rapid eGFR decline**	**Model 1**			**Model 2**			**Model 3**		
(GFR loss > 3 ml/min/1.73 m^2/year)	OR	95% CI	p value	OR	95% CI	p value	OR	95% CI	p value
All (N=1329)[a]									
HDL-C, per doubling (log2)	0.57	(0.35 - 0.91)	0.02	0.72	(0.41 - 1.28)	0.27	0.77	(0.42 - 1.39)	0.38[d]
Inactive[b] (N=731)									
HDL-C, per doubling (log2)	0.93	(0.48 - 1.77)	0.82	1.16	(0.51 - 2.63)	0.73	1.12	(0.47 - 2.70)	0.79
Active[b] (N=595)									
HDL-C, per doubling (log2)	0.39	(0.20 - 0.76)	0.01	0.48	(0.22 - 1.05)	0.07	0.52	(0.23 - 1.18)	0.12

[a]All participants were included regardless of number of eGFR measurements, because linear mixed regression allows for missing observations at ≥ 1 timepoints. Only those with ≥ 2 eGFR measurements were included in the logistic regression of rapid GFR decline. 4 missing values for physical activity.

[b]Self-reported frequency and intensity of leisure-time physical exercise as previously reported (ref 33). Active [>1-h hard physical activity a week (becoming breathless or sweaty, or exhausted) and/or >3-h light activity (without becoming breathless or sweaty)] or inactive (all others).

[c]P-value for interaction with physical activity = 0.07. [d]P-value for interaction with physical activity = 0.02. Model 1: Adjusted for sex and age. Model 2: Model 1 + BMI, low density lipoprotein (LDL), triglycerides, use of lipid-lowering drugs and alcohol consumption. Model 3: Model 2 + systolic blood pressure, fasting glucose, smoking, physical activity, waist to hip ratio, hs-CRP, albumin-to-creatinine ratio (ACR) and use of antihypertensive medications. In the logistic regression model 3 for rapid GFR decline we also included baseline GFR. Physical Activity was not included as a covariate in the stratified analyses.

Table S15. Association of baseline HDL-C levels with eGFR decline using the comined creatinine-and cystatin-based CKD-EPI equation.

	Model 1			Model 2			Model 3		
**Annual eGFR decline rate**	mL/min /1.73 m^2 per year	95% CI	p value	mL/min /1.73 m^2 per year	95% CI	p value	mL/min /1.73 m^2 per year	95% CI	p value
All (N=1627)[a]									
HDL-C, per doubling (log2)	-0.10	(-0.29 - 0.10)	0.34	-0.09	(-0.32 - 0.15)	0.45	-0.10	(-0.34 - 0.15)	0.43[c]
Inactive[b] (N=918)									
HDL-C, per doubling (log2)	-0.22	(-0.49 - 0.05)	0.11	-0.15	(-0.48 - 0.18)	0.37	-0.19	(-0.54 - 0.15)	0.27
Active[b] (N=705)									
HDL-C, per doubling (log2)	0.06	(-0.22 - 0.35)	0.66	0.01	(-0.32 - 0.34)	0.97	0.01	(-0.33 - 0.34)	0.97

**Rapid eGFR decline**	Model 1			Model 2			Model 3		
(GFR loss > 3 ml/min/1.73 m^2/year)	OR	95% CI	p value	OR	95% CI	p value	OR	95% CI	p value
All (N=1329)[a]									
HDL-C, per doubling (log2)	1.13	(0.67 - 1.90)	0.64	1.60	(0.85 - 3.00)	0.15	1.67	(0.86 - 3.24)	0.13[d]
Inactive[b] (N=731)									
HDL-C, per doubling (log2)	1.76	(0.85 - 3.65)	0.13	2.77	(1.10 - 6.96)	0.03	3.07	(1.14 - 8.25)	0.03
Active[b] (N=595)									
HDL-C, per doubling (log2)	0.68	(0.32 - 1.44)	0.31	0.88	(0.36 - 2.15)	0.78	0.84	(0.33 - 2.15)	0.71

[a]All participants were included regardless of number of eGFR measurements, because linear mixed regression allows for missing observations at ≥ 1 timepoints.
Only those with ≥ 2 eGFR measurements were included in the logistic regression of rapid GFR decline. 4 missing values for physical activity.
[b]Self-reported frequency and intensity of leisure-time physical exercise as previously reported (ref 33). Active [>1-h hard physical activity a week (becoming breathless or sweaty, or exhausted) and/or >3-h light activity (without becoming breathless or sweaty)] or inactive (all others).
[c]P-value for interaction with physical activity = 0.06. [d]P-value for interaction with physical activity = 0.02. Model 1: Adjusted for sex and age. Model 2: Model 1 + BMI, low density lipoprotein (LDL), triglycerides, use of lipid-lowering drugs and alcohol consumption. Model 3: Model 2 + systolic blood pressure, fasting glucose, smoking, physical activity, waist to hip ratio, hs-CRP, albumin-to-creatinine ratio (ACR) and use of antihypertensive medications. In the logistic regression model 3 for rapid GFR decline we also included baseline GFR. Physical Activity was not included as a covariate in the stratified analyses.
